# Interaction of Hexamethylenetetramine with Phenol–Formaldehyde Resin during Simultaneous Curing of Novolac and Resole

**DOI:** 10.1021/acsomega.5c09518

**Published:** 2026-05-01

**Authors:** Bartłomiej Milewski, Robert Antosz, Marcin Skowronek, Jadwiga Laska

**Affiliations:** † AGH University of Krakow, Faculty of Materials Science and Ceramics, Al. Mickiewicza 30, 30-059 Kraków, Poland; ‡ TECHNIFLEX Sp. z o.o., Company Branch in Mszana Dolna, ul. Spadochroniarzy 8, 34-730 Mszana Dolna, Poland; § Lerg S.A., Pustków − Osiedle 59D, 39-206 Pustków 3, Poland

## Abstract

This work is focused on the analysis of the curing process of phenol–formaldehyde resins using hexamethylenetetramine. Most of the recent studies address the curing of either liquid resole or powdered novolac resin. However, there are no published studies concerning their mixture despite the fact that this material is commonly used in the abrasive industry. In the presented study, the curing mechanism of a resole–novolac mixture in the presence of hexamethylenetetramine was investigated using simultaneous thermal analysis combined with FTIR spectroscopy. This approach enabled the qualitative and quantitative identification of volatile compounds released over a wide temperature range (40–300 °C) as well as a comparison of the curing processes of resole, novolac, and their mixtures. The results show that when both types of resins are combined, hexamethylenetetramine reacts with water produced during the curing of the resole resin, thereby altering the course of the novolac cross-linking process.

## Graphical abstract

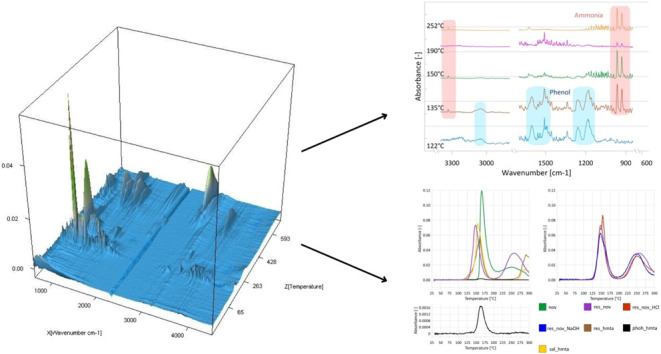

## 1 Introduction

Phenol–formaldehyde (PF) resins were the first synthetic polymer developed in the early 20th century. Their initial success was reflected in their widespread use in everyday products, electrical insulation and electronic casings. However, the introduction of cheaper and more easily processed thermoplastics and epoxy resins has led to a significant market shift. As a result, PF resins became confined to niche applications requiring exceptional thermal resistance, nonflammability, strong adhesion, and high dimensional fidelity. These applications include binders in composite materials (e.g., furniture and casting molds), insulating materials, abrasives, and friction materials.^1,2^ Two main types of PF resins are used industrially: novolacs and resoles. They differ in both their properties and their synthesis conditions. Resole resins are typically viscous liquids that can self-cure upon heating. They contain hydroxymethylene substituents in the phenolic rings, which are responsible for thermal curing. In contrast, novolac resins are usually produced in a solid, powdered form. They lack reactive functional groups and therefore require an external curing agent.^1^ Hexamethylenetetramine (HMTA) is most commonly used as a cross-linking agent due to its relatively low toxicity and ease of incorporation into resin formulations. Resole resins are synthesized via the condensation reaction of phenol and formaldehyde in an alkaline medium with an excess of formaldehyde, whereas novolacs are obtained under acidic conditions with an excess of phenol.^1,2^ The reaction schemes for resole and novolac syntheses are presented in Figure 1.

**Figure 1:**
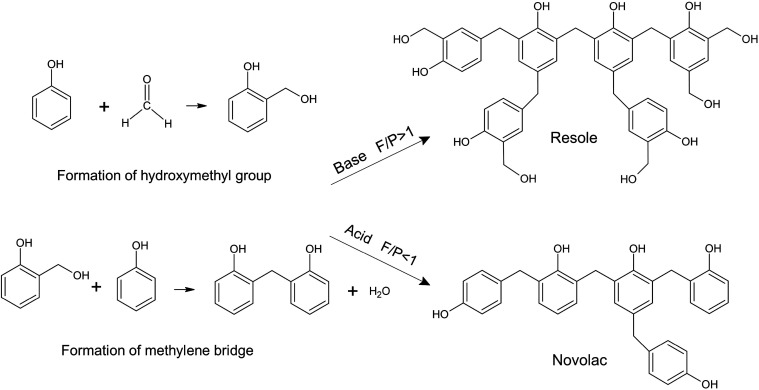
Scheme of chemical reactions leading to the formation of resole and novolac.

Despite their widespread industrial use, the factors significantly governing the curing behavior of PF resins and their influence on the final material properties remain only partially understood. Although numerous scientific studies on PF resins have been published, most focus on selected aspects of composite material production.^3–16^ Another group of scientific articles addresses the curing process itself. The most comprehensive and systematic investigations of PF resin curing were conducted by Solomon and co-workers.^17–30^ These works provide an in-depth analysis of novolac cross-linking and also address resoles, furfuryl resins, polyimides, and their mixtures. Of particular importance is the work by Zhang et al.,^22^ who employed NMR spectroscopy to monitor chemical reactions during novolac cross-linking. By using ^13^C and ^15^N-labeled hexamethylenetetramine, the authors demonstrated that the amount of cross-linking agent significantly influences the reaction pathway. They identified ortho- and parahydroxybenzylamines and benzoxazines (shown in Figure 2) as key intermediates. Subsequent studies further examined the reactions of these intermediates using NMR techniques.^23–25,28,29^

**Figure 2:**
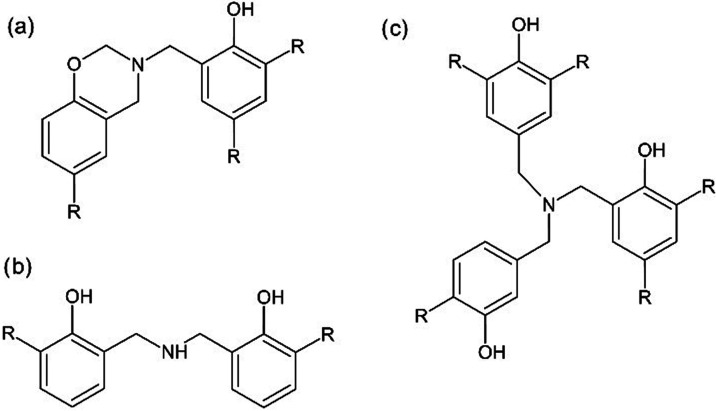
Most common intermediates of novolac cross-linked with hexamethylenetetramine: (a) benzoxazines, (b) dibenzylamines, and (c) tribenzylamines.

The manner in which phenolic rings are linked during both resin synthesis and curing has a profound effect on the final properties of the cured PF materials. Phenolic rings are typically connected via methylene (−CH_2_−) bridges, forming o-o’, o-p’, and p-p’ linkages in relation to hydroxyl groups, as well as o-o’ oxidimethylene bridges. Hu et al.^31^ employed temperature-variable FTIR and TG-MS analyses to investigate the curing of resole resins. Their studies showed that the temperature strongly influences the sequence of bridge formation. Between 90 and 120 °C, the formation of p-p’, o-o’, and ether bridges was favored. In the range of 120–160 °C, p-p’, o-o’, and o-p’ linkages predominated, while at 160–190 °C, cleavage of oxidimethylene bridges was observed. Other studies have shown that resole curing proceeds via a quinone methide intermediate^32,33^ and that phenoxy bridges may also form during this process.^33^ The behavior of a furfuryl alcohol-novolac system was investigated by Zhang et al.^34^

The objective of the present study was to elucidate the chemical reactions and interactions occurring during the curing of a mixture of resole, novolac, and hexamethylenetetramine. Comprehensive investigations were conducted using simultaneous thermal analysis (STA) coupled to Fourier Transform Infrared (FTIR) spectroscopy. Measurements were performed for individual resins as well as their mixtures. Additionally, resole–novolac mixtures containing small amounts of hydrochloric acid or sodium hydroxide were examined, as mineral fillers commonly added to industrial resin formulations can alter the system pH. These experiments were therefore designed to assess the influence of minor pH variations on the curing kinetics. For comparative purposes and to facilitate interpretation of the results, analogous STA-FTIR studies were also performed on simple chemical analogues, namely, salicyl alcohol and phenol in combination with hexamethylenetetramine. The results indicate that the curing processes of resole and novolac are interdependent on their mixture. The presence of resole initiates additional reactions of hexamethylenetetramine, significantly influencing the curing process by altering reactant concentrations, increasing heat release, and generating new reactive sites. With the exception of the work by Chow,^35^ no systematic studies comparing the curing behavior of this widely used mixture in organic resin-bonded abrasives production, have been reported.

## 2 Materials and Methods

Resole, novolac, phenol, and hexamethylenetetramine (HMTA) were purchased from Lerg S.A. (Poland). The selected commercial resole contained approximately 20 wt% of phenol and 6 wt% of water, while the novolac contained 14 wt% of HMTA. Hydrochloric acid and sodium hydroxide were purchased from Avantor Performance Materials Poland S.A. (Gliwice, Poland). Salicyl alcohol was purchased from Merck (Germany).

Mixtures of novolac, resole, HMTA, and other components were prepared by mechanical mixing until a homogeneous composition was obtained. All measurements were performed immediately after the sample preparation.

In total, nine different samples were analyzed and grouped as follows:

(1) reference systems
  - resole (**res**);
  - novolac powder containing 14% of hexamethylenetetramine by weight (**nov**);
  - resole mixed with hexamethylenetetramine at a weight ratio 1:0.15 (**res_hmta**);
(2) investigated system
  - resole mixed with novolac at a weight ratio 1:2 (**res_nov**);
(3) pH influence evaluation systems
  - resole mixed with novolac and 0.1 M HCl at a weight ratio 1:2:0.02 (**res_nov_HCl**);
  - resole mixed with novolac and 0.1 M NaOH at a weight ratio 1:2:0.02 (**res_nov_NaOH**);
(4) low-molecular-weight model systems
  - salicyl alcohol mixed with hexamethylenetetramine at a weight ratio 2:1 (**sal_hmta**);
  - phenol mixed with hexamethylenetetramine at a weight ratio 2:1 (**phoh_hmta**);
(5) high-pressure experiments:
  - novolac powder containing 14% of hexamethylenetetramine by weight with the addition of 5% wt. of water (**nov_water**).

The resole-to-novolac 1:2 ratio was selected because similar proportions are commonly used in the abrasive industry. The resole-to-HMTA ratio corresponded to the HMTA content in the commercial novolac sample.

Simultaneous thermal analysis (STA) was performed using a Netzsch STA 449 F3 Jupiter instrument coupled with an FTIR spectrometer (Bruker Tensor 27 equipped with a liquid-nitrogen-cooled mercury cadmium telluride detector) to enable in situ analysis of thermally induced reactions. An empty crucible was used for baseline correction in STA measurements, while the FTIR background spectrum was recorded for gas composition in the transfer line prior to heating. Samples with weights ranging from 7 to 13 mg were placed in open corundum crucibles. They were heated from 40 to 300 °C at a rate of 10 °C·min^–1^, and volatile products were monitored in real time using FTIR spectroscopy. The transfer line and the detector chamber were maintained at 250 °C to prevent condensation or deposition of volatile compounds. FTIR spectra were processed by using Opus software (version 7.2) and Spectragryph software (version 1.2.16.1). The volatiles were identified with the use of Bruker NIST/EPA Vapor Phase Library. Following identification, each compound was assigned a characteristic absorption band. The temporal evolution of the normalized band intensity, plotted as a function of temperature, was used to describe the kinetics of volatile release. These plots are hereafter termed release curves. The complete list of absorption bands selected for kinetic analysis is presented in Table 1.

**Table 1:** FTIR Bands Characteristic of the Volatile Components Released during the Cross-Linking Process were Selected to Determine Their Release Curves^a^

	characteristic FTIR bands of the volatiles [cm^–1^]				
sample	NH_3_	PhOH	water	HMTA^36^	SAL
nov	966		3853	2958	
res		1184	3853		
res_nov	966	1184	3853	2958	
res_nov_HCl	966	1184	3853		
res_nov_NaOH	966	1184	3853		
res_hmta	966	1184	3853	2958	
phoh_hmta	931^b^	1184		2958	
sal_hmta	966		3853	2958	750

a Abbreviations: SAL – salicyl alcohol, PhOH – phenol.

b In the case of phoh_hmta, a weaker band at 931 cm^–1^ was used instead of the 966 cm^–1^ one, because the latter’s intensity was affected by the intensity of the HMTA–attributed band at 1011 cm^–1^.

To evaluate the influence of water on novolac cross-linking, high-pressure differential scanning calorimetry (DSC) measurements were conducted using a NETZSCH DSC 214 Polyma instrument. High-pressure steel crucibles were employed for the samples, with a pierced aluminum crucible as the reference. The samples were heated at a rate of 10 °C·min^–1^ from −40 or 10 °C to 300 or 320 °C, depending on the experimental conditions.

## 3 Results

### 3.1 Simultaneous Thermal Analysis Coupled with FTIR Spectroscopy

#### 3.1.1 Novolac–HMTA System

The commercial novolac used in this study contains 14 wt% of hexamethylenetetramine. According to the manufacturer, neat novolac exhibits only a second-order transition associated with the glass transition at approximately 57 °C (Supporting Information, Figure S1). Upon addition of HMTA, a pronounced exothermic effect appears, with a maximum near 150–160 °C, accompanied by a weaker and broader peak extending up to about 300 °C (Supporting Information, Figure S2).

The TG curve obtained during STA-FTIR measurements (Figure 3) shows that novolac exhibits no significant mass loss up to 100 °C. Between 100 and 150 °C, only a minor mass loss of approximately 0.5% is observed, followed by a more pronounced decrease of 2.1% up to 170 °C, with a maximum mass-loss rate of 0.21%·min^–1^ at 159 °C (Figure 4). The total mass loss reaches 5.3% at 300 °C. These results indicate that novolac cross-linking in the presence of HMTA occurs predominantly in the range 150–170 °C, which is confirmed by the DSC results and FTIR analyses. The HMTA release curve (Figure 5) shows that hexamethylenetetramine begins to volatilize at approximately 90 °C. It is the only volatile compound detected below 155 °C (Figure 6) and is therefore responsible for the observed mass loss in this temperature range. HMTA-related IR bands are barely visible at 100 °C but increase significantly between 120 and 160 °C, reaching maxima at 137 and 164 °C. Above 180 °C, HMTA bands weaken and disappear completely at 190 °C. Literature data suggest HMTA sublimation temperature above 150 °C^37^ or even 220 °C,^38^ whereas the present results indicate that HMTA volatilization in the novolac̵–HMTA mixture occurs over a broader and lower temperature range (90–200 °C).

**Figure 3:**
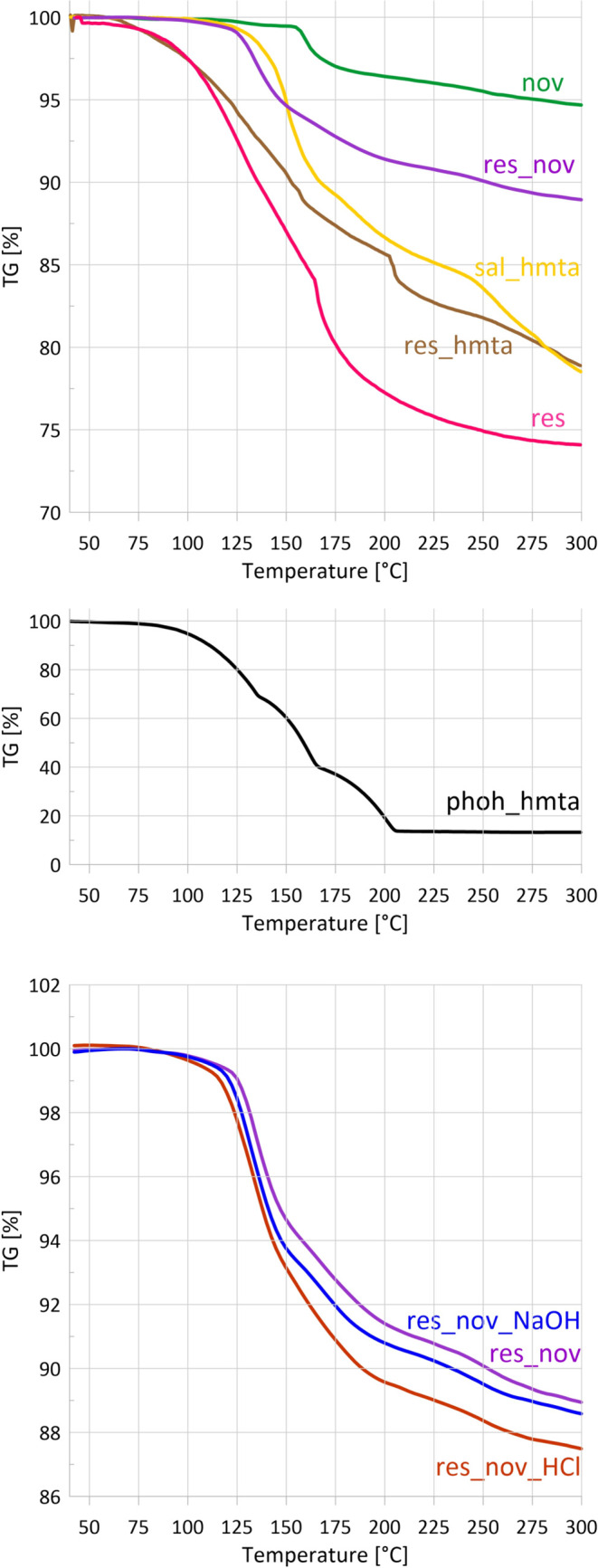
TG curves of all samples collected during the STA-FTIR measurements. Mass loss occurs due to the evaporation of both volatile components of the samples and products of curing reactions. Mixtures of resole and novolac are shown in a separate chart, in order to showcase the effect of the addition of acid and base. Sample phoh_hmta was plotted separately due to a different TG curve range.

**Figure 4:**
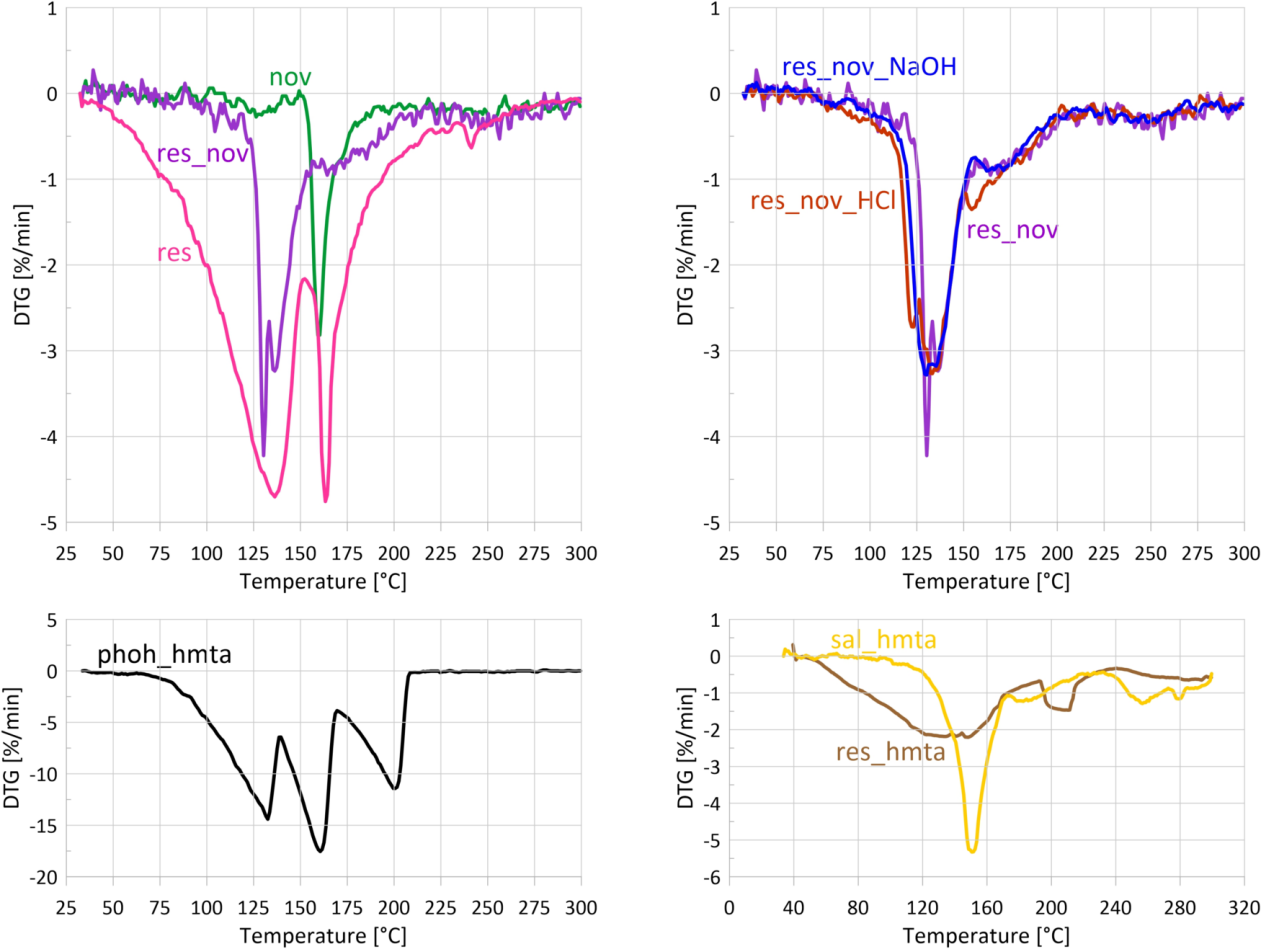
DTG curves of all samples were based on STA-FTIR measurements. DTG maxima can indicate evaporation of volatile components of the samples or release of curing byproducts, as in the case of novolac. Samples are plotted in 4 charts in order to increase readability.

**Figure 5:**
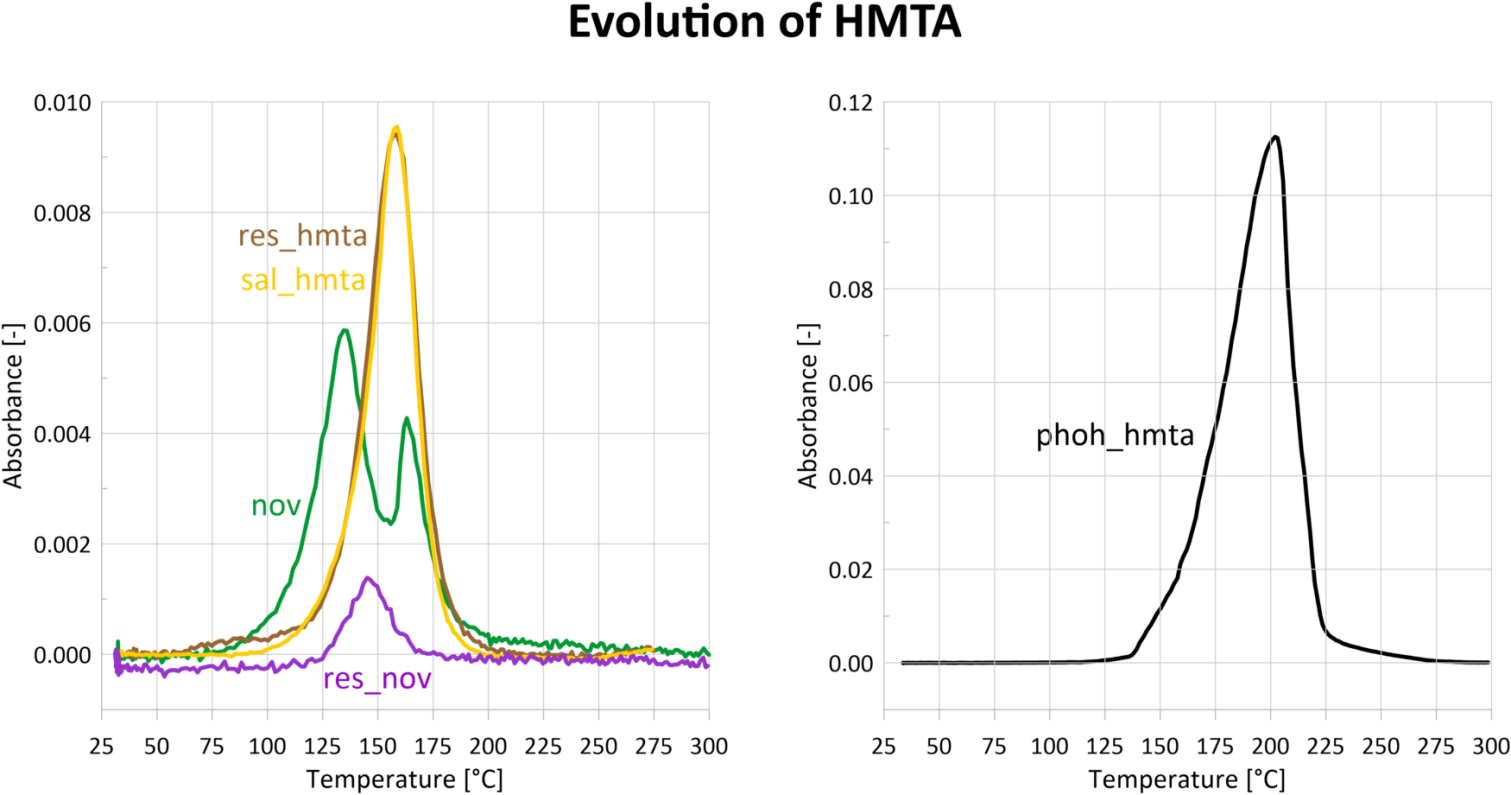
Release curves of HMTA (absorbance at 2958 cm^–1^) evolving during the STA-FTIR measurements. During cross-linking, most of the HMTA should be consumed in the reaction. Its high emission during heating of the phenol/hmta mixture indicates that no cross-linking occurs.

**Figure 6:**
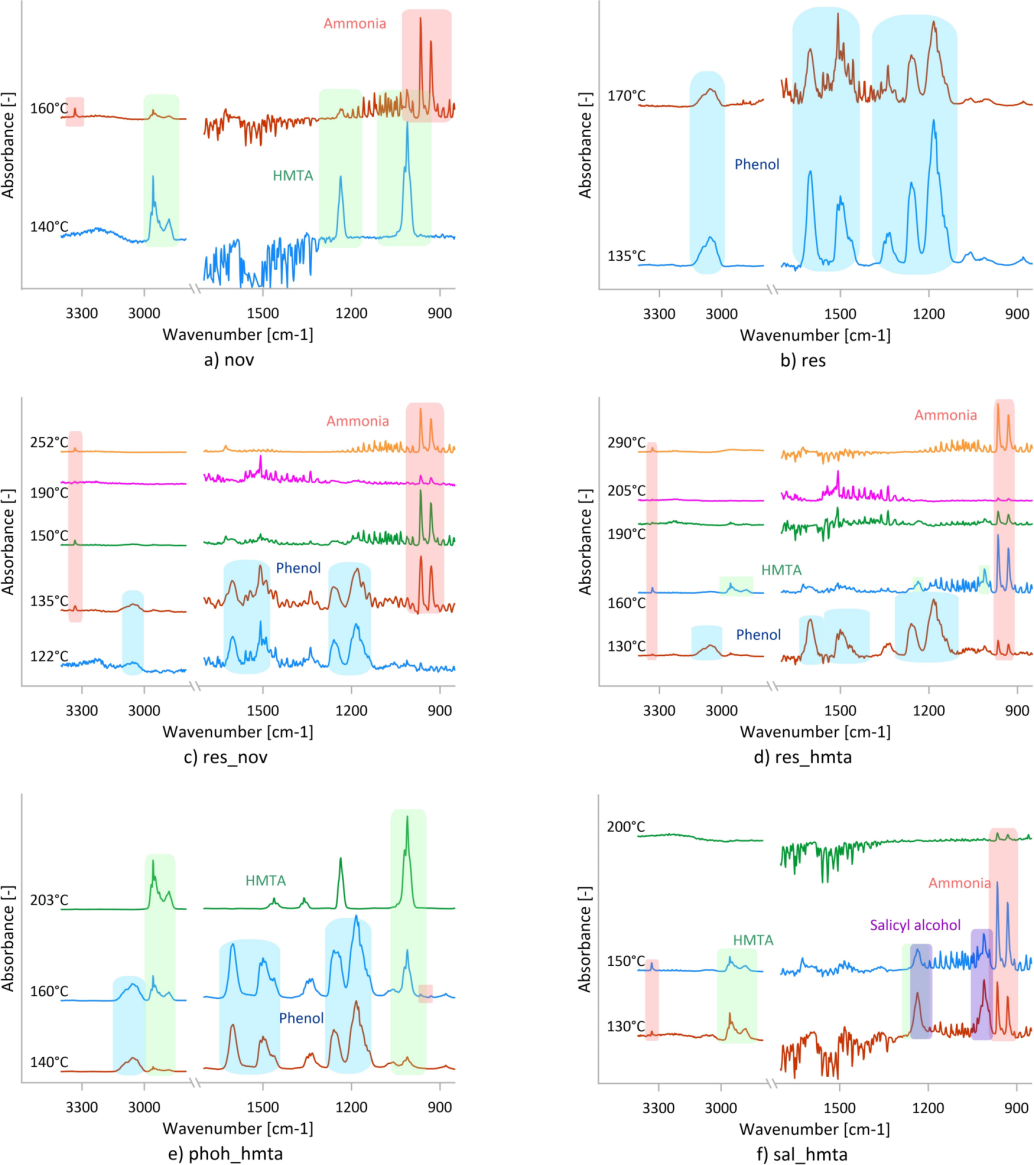
Chosen FTIR spectra of the volatiles evolving from the samples at specific temperatures (a, novolac; b, resole; c, mixture of resole and novolac; d, mixture of resole and HMTA; e, mixture of phenol and HMTA; f, mixture of salicyl alcohol and HMTA) during STA-FTIR measurements. Offsets were applied to increase clarity.

Ammonia, a known byproduct of novolac cross-linking with HMTA,^39^ is identified by characteristic IR bands at 931, 966, and 3330 cm^–1^. Weak ammonia signals appear at approximately 157 °C, reach a maximum at 167 °C, decrease above 180 °C, and increase again above 210 °C with a second maximum near 250 °C (Figures 6 and 7). This two-stage behavior indicates that, after the main cross-linking event, thermally labile nitrogen-containing intermediates persist in the cured resin and decompose at elevated temperatures. The aggregated mass loss of 2.9% between 152 and 190 °C (Figure 3) confirms that this temperature range corresponds to intensive cross-linking. Above 280 °C, ammonia emission becomes negligible, indicating complete HMTA consumption and termination of the curing process. Neither formaldehyde nor phenol was detected in the volatiles, and no resin decomposition was observed over the entire temperature range. The novolac/HMTA system, therefore, provides a well-defined reference: curing initiates sharply above 150 °C, proceeds with ammonia release, and leaves behind nitrogen-containing byproducts that undergo slow secondary decomposition at higher temperatures.

**Figure 7:**
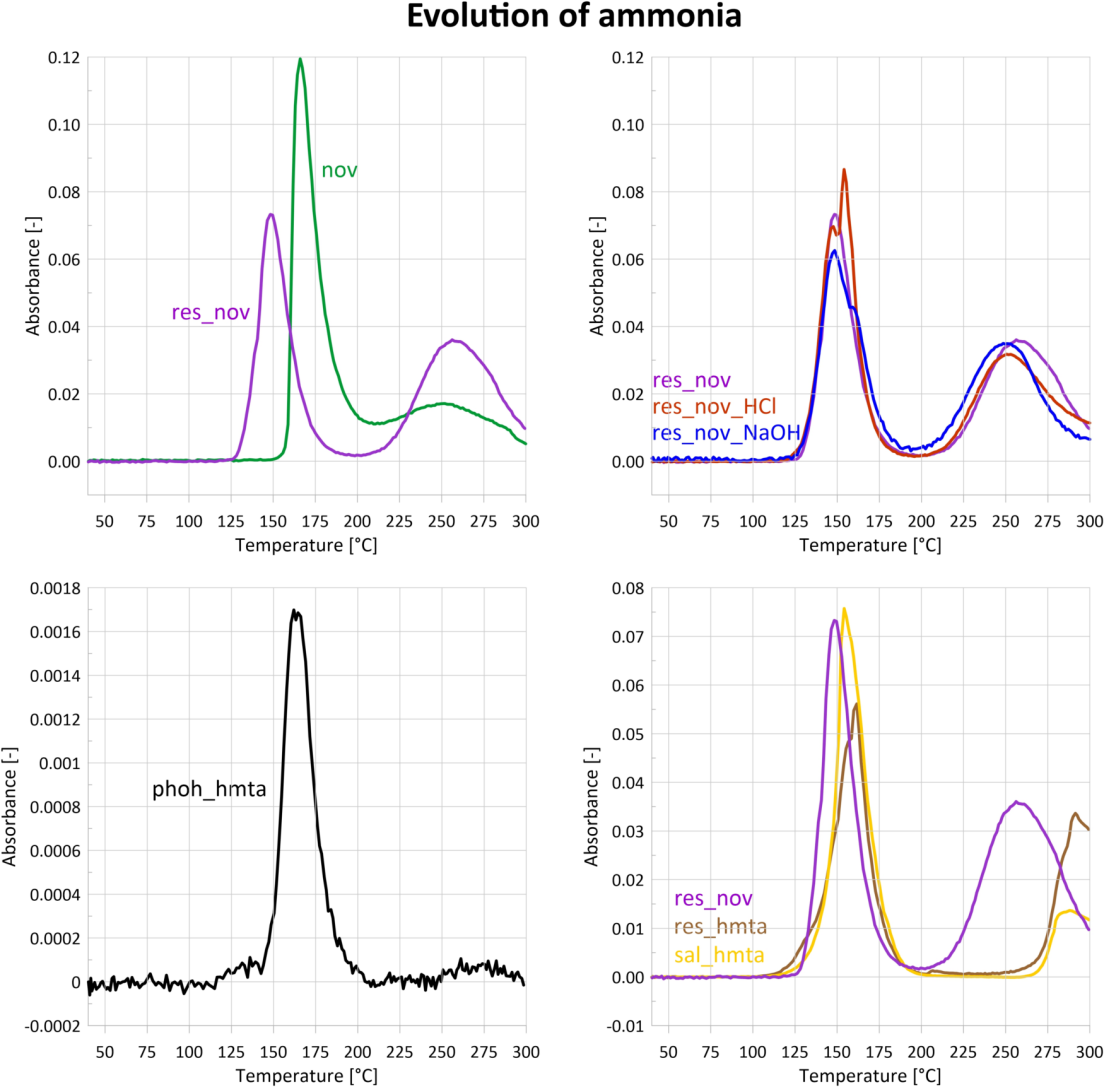
Release curves of ammonia (absorbance at 966 and 931 cm^–1^ for sample phoh_hmta) evolving during STA-FTIR measurements. Its evolution can mark cross-linking with the use of HMTA or the decay of nitrogen-containing intermediates. Samples were plotted in 4 charts in order to increase clarity.

#### 3.1.2 Resole

The commercial resole resin contains approximately 6 wt% water and 20 wt% of phenol. TG analysis (Figure 3) shows a total mass loss of 23% up to 200 °C, reflecting the evaporation of low-molecular components and the release of water formed during curing. Interpretation of the DSC curve is challenging due to the overlap between endothermic evaporation of phenol and water and the exothermic curing reaction (Figure 9). Nevertheless, the main curing exotherm is visible at around 206 °C. Additional high-pressure DSC measurements (Supporting Information, Figure S4) confirm that this effect originates from curing, as evaporation is suppressed. Nevertheless, the DTG maxima are at approximately 135 and 165 °C.

DTG analysis (Figure 4) reveals two distinct mass-loss-rate maxima at 135 and 165 °C (3.6 and 4.0%·min^–1^, respectively), which clearly distinguish two overlapping processes: volatilization of phenol and progressive network formation accompanied by water release.

STA-FTIR spectra (Figures 6 and 8) show that phenol is released between 60 and 200 °C, with a maximum at a temperature of 135 °C, coinciding with the first DTG maximum (Figure 4) and a broad endothermic DSC effect (Figure 9). Phenol bands disappear above 200 °C.

**Figure 8:**
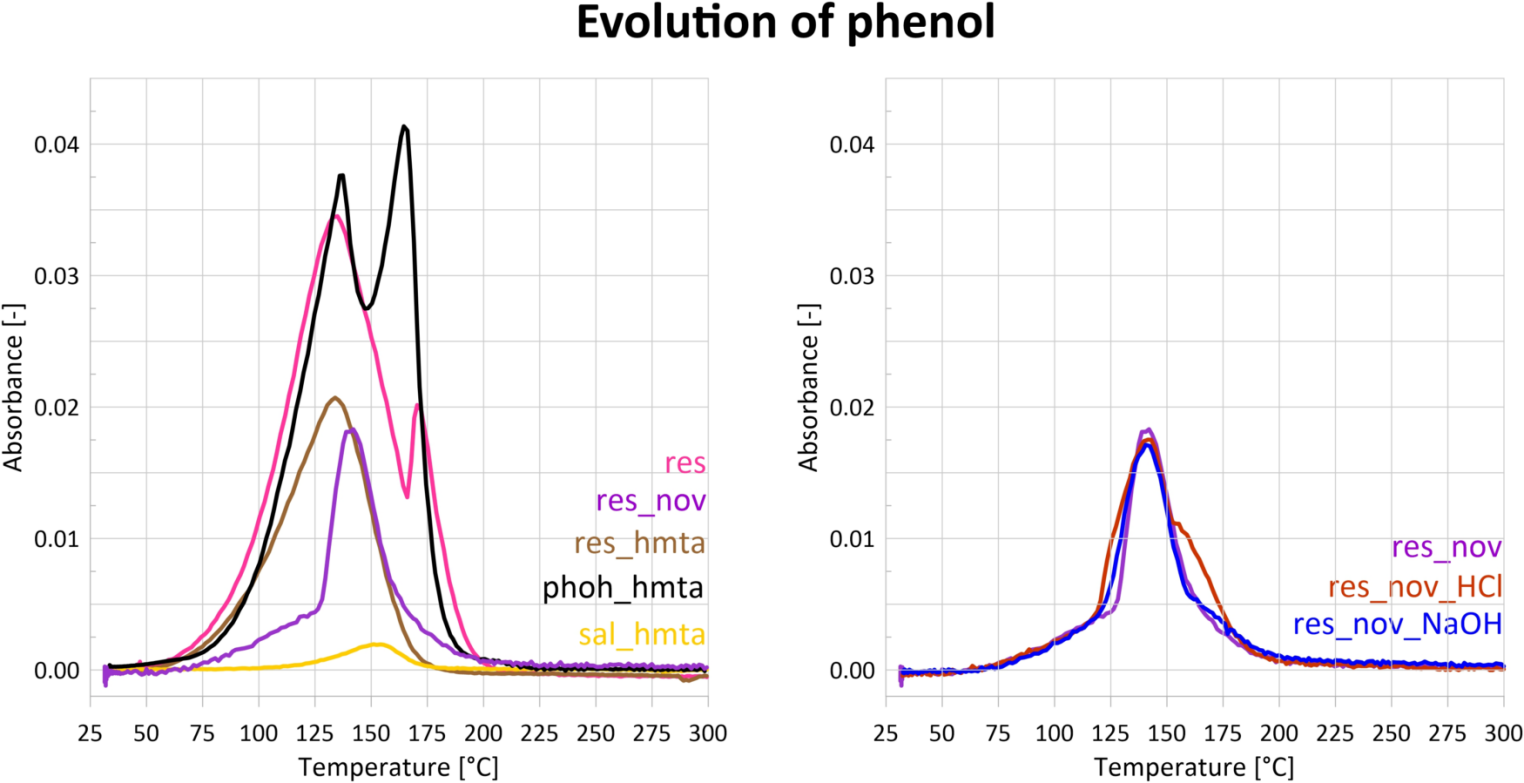
Release curves of phenol (absorbance at 1184 cm^–1^) during STA-FTIR measurements. For sample sal_hmta, a release curve of salicyl alcohol is presented instead (absorbance at 750 cm^–1^). Phenol contained within resole can evaporate or be incorporated into the resin structure. The much lower phenol release in the case of resole and novolac mixtures means that a significant amount of it reacts with the resins.

Water vapor is released in the wide temperature range (Figure 10) with onset at ∼110 °C and maximum between 163 and 170 °C, corresponding to the second DTG peak (Figure 4) and the second endothermic effect in the DSC curve (Figure 9).

**Figure 9:**
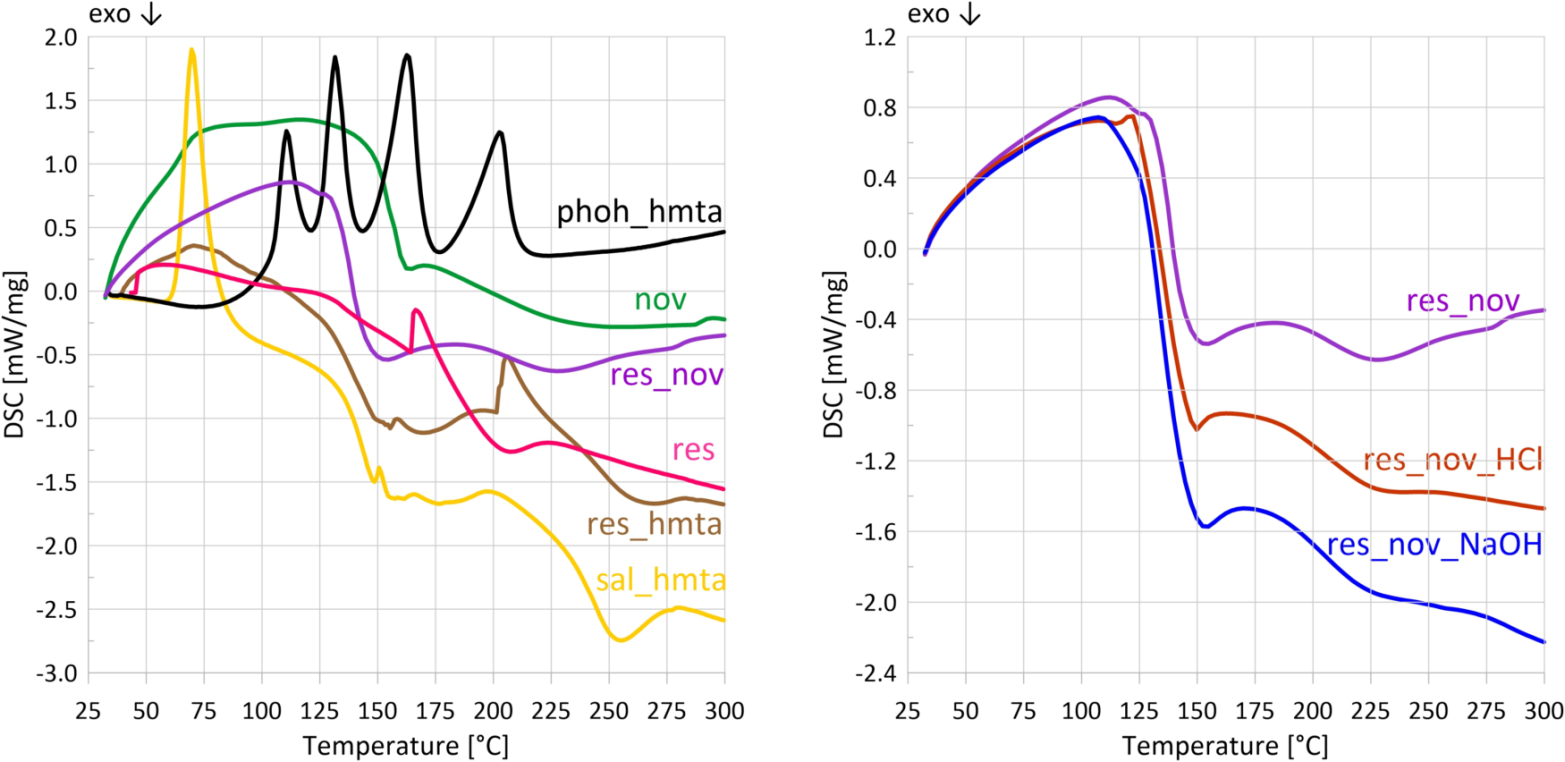
DSC curves from STA_FTIR measurements for all of the samples. Most curves contain high baseline shift, yet characteristic effects are still visible. Exothermic effect originating from curing is present in all samples besides phoh_hmta.

Throughout the entire temperature range, no other organic substances were detected, indicating that resole curing proceeds without the release of free formaldehyde. In comparison with novolac, resole exhibits a much broader curing temperature range, extending from below 100 to above 200 °C, and produces substantial amounts of volatiles during heating.

#### 3.1.3 Resole–Novolac Mixture

The thermal behavior of the novolac–resole mixture (Figure 9) combines features of both individual resins, yet differs fundamentally from both of them. The DSC curve resembles that of novolac but exhibits two exothermic peaks, the first between 140 and 164 °C, with a maximum at 155 °C, and the second in the range of 200–260 °C, with a maximum at 230 °C. The fastest mass loss (Figures 3 and 4) occurs between 120 and 152 °C, with the maximum rate of 0.2%·min^–1^ at 135 °C. The total mass loss is ∼7%.

FTIR spectra (Figures 7 and 8) reveal simultaneous emission of phenol, water, ammonia, and traces of HMTA, reflecting contributions from both components. Phenol release begins at 70 °C and reaches a maximum at 141 °C, closely resembling the behavior of resole. Water vapor appears at ∼125 °C, and the emission shows two maxima at approximately 145 and 180 °C (Figure 10). Ammonia – characteristic of novolac curing with HMTA - begins to evolve at lower temperatures than in neat novolac, i.e., at 130 °C (Figure 7), reaches a maximum at 148 °C, and disappears at 200 °C. Similar to novolac, the secondary emission starts at 200 °C. However, in the mixture, the first ammonia emission is markedly weaker than in novolac, whereas the secondary emission is considerably stronger. This indicates that in the presence of resole, the curing reaction favors the formation of nitrogen-containing intermediates decomposing at higher temperatures. Only traces of HMTA were present in the volatiles (Figure 5). These observations demonstrate that the simultaneous presence of novolac, resole, and HMTA does not merely superimpose two independent curing processes. Instead, the interaction between the components fundamentally alters the reaction pathway, leading to earlier initiation of HMTA-mediated cross-linking and to the accumulation of thermally labile nitrogen-containing species. This behavior points toward a key role of water and hydroxymethylene groups in modifying the mechanism of HMTA activation.

**Figure 10:**
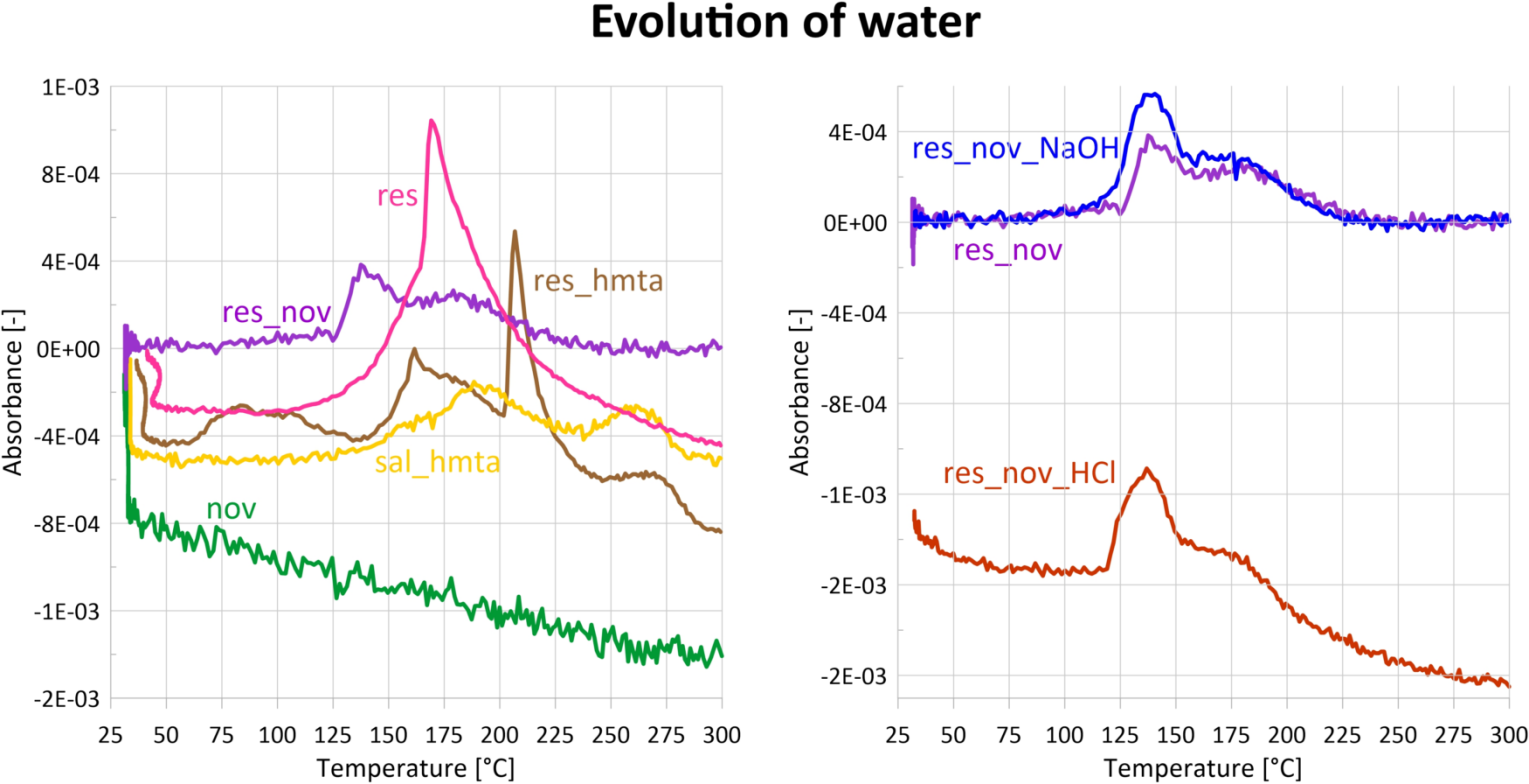
Release curves of water (absorbance at 3853 cm^–1^) during STA-FTIR measurements. Evaporating water can have two sources: it is present in the samples or is produced as a result of curing via hydroxymethylene groups.

#### 3.1.4 Low-Molecural-Weight Model Systems

Reference mixtures containing resole–HMTA, phenol–HMTA, and salicyl alcohol–HMTA were examined to clarify the individual reaction pathways. Phenol serves as a molecular analogue of novolac repeating units, whereas salicyl alcohol represents a simplified model of hydroxymethylene-substituted phenolic rings characteristic of resoles.

##### 3.1.4.1 Resole–HMTA System (res_hmta)

The thermal behavior of res_hmta differs markedly from that of the neat resole. TG and DTG curves (Figures 3 and 4) show mass loss starting at about 50 °C and continuing up to 300 °C. The highest mass loss rate (2.2%·min^–1^) occurs between 120 and 150 °C, corresponding to ∼7% mass loss. A second, weaker DTG maximum is observed at 210 °C, followed by gradual mass loss up to 300 °C, resulting in a total mass loss of 21%.

FTIR spectra of the evolved volatiles (Figures 6 and 8) indicate phenol release between 50 and 175 °C, with a maximum at 135 °C, consistent with the observations of the neat resole. Water vapor is released in four stages (Figure 10). The first traces appear at 60 °C, and the release continues up to 140 °C, with a maximum at 90 °C and accounting for 9% mass loss. The second stage between 140 and 200 °C contributes an additional 5% mass loss, with a distinctive and narrow maximum at 163 °C (Figures 3 and 4). Intensive water release is observed again between 200 and 225 °C (3% mass loss), followed by weaker release up to 300 °C. IR bands originating from ammonia (Figure 6) are observed in two temperature ranges of 120–200 °C and 250–300 °C, with maxima at 160 and 290 °C, respectively. The second region of ammonia release coincides with a 3% mass loss (Figure 3).

Interpretation of the DSC curve for res_hmta (Figure 9) remains ambiguous due to the overlap of endothermic melting and evaporation effects with exothermic chemical reactions. HMTA appeared in the volatiles in the range 70–190 °C, with a maximum emission at 160 °C (Figure 5).

##### 3.1.4.2 Phenol–HMTA System (phoh_hmta)

In contrast to the resole-containing system, heating phenol mixed with HMTA results exclusively in endothermic effects. The DSC curve (Figure 9) exhibits four endothermic peaks with maxima at 113, 130, 162, and 205 °C, and no exothermic effects, which suggests the absence of chemical reactions between phenol and HMTA. The first endothermic effect corresponds to the melting of the mixture.^40^ The subsequent three endothermic effects are accompanied by extensive mass loss, reaching about 87% at 300 °C (Figure 3), leaving char above 210 °C predominantly. The DTG curve (Figure 4) shows three mass loss rate maxima at 133, 161, and 200 °C. FTIR spectra of evolved volatile substances (Figure 6) consist mainly of phenol and HMTA bands across the entire temperature range, with only weak ammonia-related signals above 150 °C. These observations indicate that phenol does not participate in HMTA-mediated cross-linking under the applied conditions. HMTA remains thermally stable over the entire temperature range of 40–300 °C and does not decompose into amines, ammonia, hydrogen cyanide, or other products suggested in the literature.^37^ Phenol is released predominantly in the temperature range of 100–180 °C (Figure 8), exhibiting two intensity maxima at 140 and 162 °C. The bimodal phenol release may be related to strong interactions between phenols and amines, such as hydrogen bonding or ammonium phenolate-like structures, which are favored for higher-order amines due to steric effects.^41^

##### 3.1.4.3 Salicyl Alcohol–HMTA System (sal_hmta)

The DSC curve of sal_hmta (Figure 9) shows a strong endothermic peak with a maximum at 70 °C, attributed to the melting of the alcohol. The melting point of pure salicyl alcohol (83–87 °C) is likely lowered by the presence of HMTA. The TG curve (Figure 3) confirms that no mass loss occurs up to 100 °C, which supports assigning this peak to a physical transition. The DTG curve (Figure 4) reveals three mass loss regions: 125–170 °C (maximum at 150 °C, 5.3%·min^–1^), 175–200 °C (1.25%·min^–1^), and 250–300 °C (two maxima at 255 and 279 °C, 1.2%·min^–1^). The largest mass loss of 9% occurs in the first temperature range, and the total mass loss reaches around 22% at 300 °C, closely matching the behavior of res_hmta (21%) and differing markedly from phoh_hmta (87% of mass loss at 300 °C). Also, the DSC curves of sal_hmta and res_hmta are analogous (Figure 9).

FTIR spectra (Figure 6) show the evolution of four volatile substances: salicyl alcohol, ammonia, HMTA, and water vapor. Salicyl alcohol evaporates between 100 and 180 °C, with a maximum at 152°, corresponding to a significant mass loss visible in the TG curve (Figure 3). Ammonia is released in the temperature range of 120–200 °C, with a maximum at 155 °C (Figure 7), confirming that a chemical reaction occurs within the system. This is further supported by the presence of an exothermic DSC effect at the same temperature and a DTG maximum at 151 °C. A second ammonia release region starts at 270 °C and extends beyond 300 °C. Water is released in two temperature ranges, 125–240 °C and 240–285 °C (Figure 10), with maxima near 190 and 265 °C. The first maximum should be attributed to the curing via hydroxymethylene groups, as it occurs in the same temperature range as chemically similar resole curing. HMTA volatilization occurs mainly between 100 and 180 °C (Figure 5), similarly to the res_hmta system.

All thermal effects observed in the STA studies are summarized and described in Table 2.

**Table 2:** List of All Observed Transitions in the STA-FTIR Measurements

sample	transition	DSC peak [°C]	TG range [°C] (weight loss [%])	DTG peak [°C]	volatile, release temp. range [°C] (max. release temp. [°C])
nov	glass transition	∼70			
	cross-linking	∼162 exo	152–190 (2.9)	160	ammonia, 157–210 (167)
	decomposition of intermediates		210–300 (1.6)		ammonia, 210–300 (∼250)
	HMTA evaporation				HMTA, 90–200 (137 and 164)
res	curing with hydroxymethylene	∼206 exo	40–200 (23.5)	165	water, 110–300 170
	water evaporation	167			
	phenol evaporation	∼130		125	phenol, 60–200 (135 and 172)
res_nov	cross-linking with HMTA	∼152	100–160 (5.9)	135	ammonia, 130–200 (148)
	phenol evaporation				phenol, 70–200 (141)
	curing with hydroxymethylene		160–220 (3.0)	170	water, 125–220 (145 and 180)
	decomposition of intermediates		220–280 (1.7)	∼250	ammonia, 210–300 (260)
res_nov HCl	cross-linking with HMTA	∼150	70–152 (7.1)	135	ammonia, 120–190 (148 and 153)
	phenol evaporation				phenol, 70–210 (143 and 156)
	curing with hydroxymethylene		152–200 (3.3)	157	water, 119–200 (138 and ∼180)
	decomposition of intermediates		∼220–275 (1.3)	252	ammonia, 200–300 (253)
res_nov NaOH	cross-linking with HMTA	∼152 exo	80–157 (6.7)	132	ammonia, 126–195 (148)
	phenol evaporation				phenol, 80–200 (142)
	curing with hydroxymethylene		157–212 (2.8)	170	water, 90–225 (140 and 175)
	decomposition of intermediates	∼225 exo	214–270 (1.4)	250	ammonia, 200–290 (250)
res_hmta	water evaporation		50–193 (14.0)	120–150	water contained within resole, ∼65–120
	phenol evaporation				phenol, ∼60–180 (135)
	cross-linking with HMTA	∼150 exo			ammonia, 110–190 (162)
	curing with hydroxymethylene	∼170 exo			water, 143–200 (163)
	water evaporation	208 endo	193–215 (2.8)	208	water trapped in the resin, 202–230 (207)
	secondary curing with hydroxymethylene	∼260 exo	242–300 (3.2)	above 250	water, 250–290 (∼275)
	decomposition of intermediates				ammonia, above 260 (293)
phoh_hmta	melting	113 endo			
	curing with HMTA				trace amounts of ammonia, 144–200 (170)
	phenol evaporation	133, 164 endo	50–207 (86)	133, 161	phenol, 50–210 (137 and 166)
	HMTA sublimation/evaporation	205 endo		201	HMTA, 100–230 (204)
sal_hmta	melting	71 endo			
	salicyl alcohol evaporation				salicyl alcohol, 100–180
	curing with HMTA	∼155 exo	100–173 (10.4)	152	ammonia, 120–190 (154)
	HMTA evaporation				HMTA, 124–180
	curing with hydroxymethylene	∼180 exo	175–217 (3.6)	180	water, 135–240 (190)
	secondary curing with hydroxymethylene	253 exo	240–270 (3.1)	256	water, 240–280 (265)
	intermediates decomposition/residual HMTA decomposition		270–300 (2.8)	280	ammonia, above 267 and carbon monoxide, 247–274 (257)

### 3.2 High-Pressure Differential Scanning Calorimetry

The most consistent interpretation of all observations is that water is the key factor responsible for lowering the effective curing temperature in resole-containing systems, primarily by promoting HMTA hydrolysis and generation of reactive intermediates (formaldehyde and aminomethyl species).^38^ High-pressure DSC measurements were performed to evaluate the influence of retained water on the curing behavior of the novolac-based systems. Under open-crucible STA–FTIR conditions, free water present in commercial novolac powder can evaporate before the curing temperature is reached, reducing its ability to participate in HMTA hydrolysis. In resole-containing systems, however, water is present in the formulation and is additionally produced during curing via hydroxymethylene groups, making it available precisely at the temperature of early HMTA activation. It is worth mentioning that resole has much higher affinity toward water than novolac, due to the presence of hydroxymethylene substituents.

The hypothesis presented above is directly supported by high-pressure DSC experiments (Figure 11). By using sealed steel closed crucibles, evaporation of water was suppressed, allowing reactions to proceed in the presence of moisture. The following samples were examined: novolac (nov), novolac with 5% added water (nov_water), the resole–novolac mixture (res_nov), and phenol with HMTA (phoh_hmta). For comparison, novolac was also tested in a pierced aluminum crucible.

**Figure 11:**
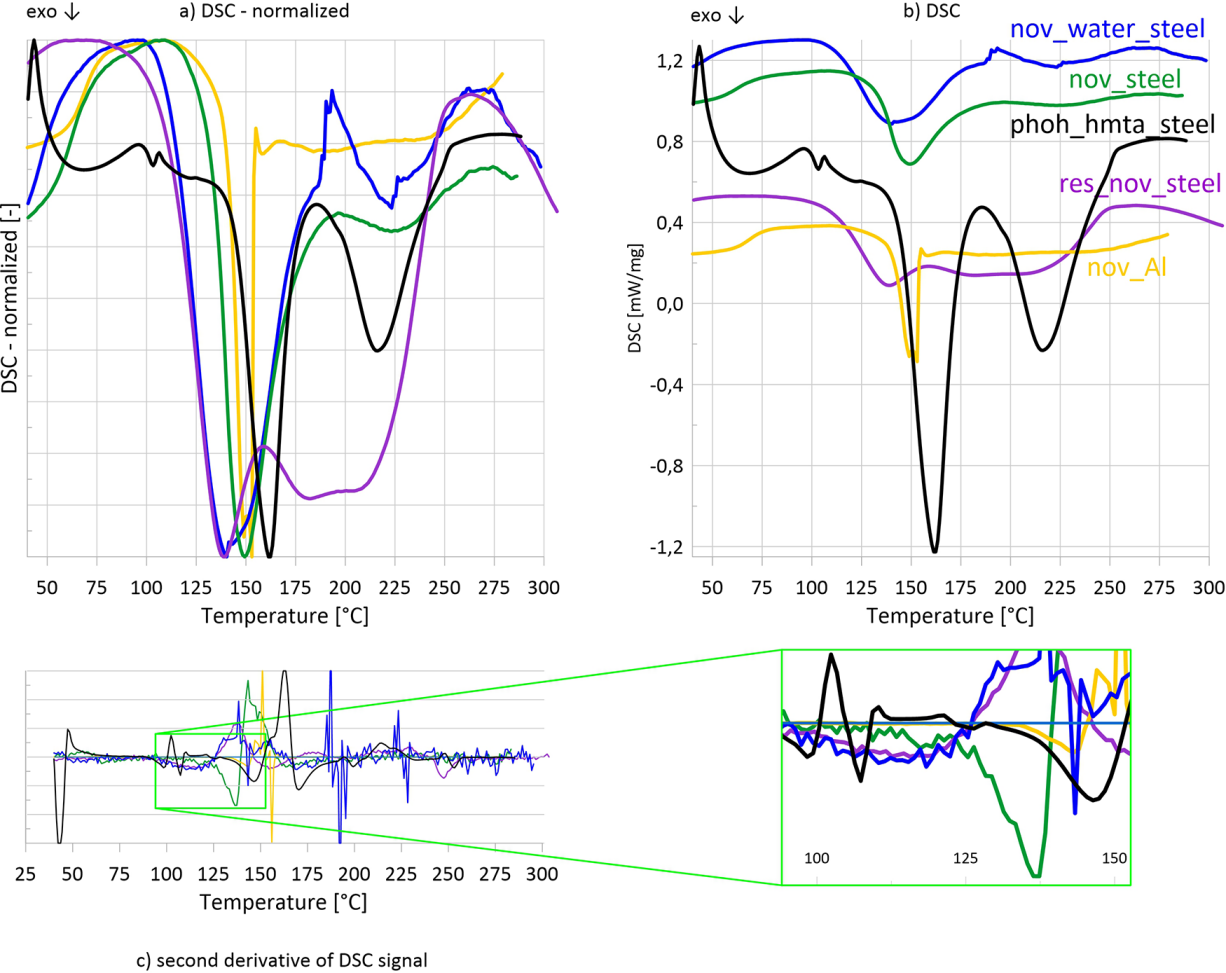
DSC curves obtained in high-preassure measurements in steel crucibles and a closed pierced aluminum crucible (sample nov_Al) (a, b, c). Evaporation of volatiles was suppressed. Addition of water to novolac decreases cross-linking temperature in the same way as the addition of resole.

For novolac in a steel crucible (nov_steel), the exothermic curing effect begins at 137 °C, reaching a maximum at 149 °C. In the pierced aluminum crucible, where moisture can escape, the onset and peak temperatures are shifted upward to ∼143 and 151 °C, respectively. This small but systematic difference indicates that even the trace amounts of water present in commercial novolac influence the curing kinetics when they are retained in the system.

When evaporation is suppressed, and water is retained within the sample, adding 5 wt% water to novolac (nov_water) shifts the curing onset from 137 to 118 °C and moves the maximum from ∼149 to 140 °C, essentially reproducing the behavior also observed for the resole–novolac (res_nov) mixture measured under the same conditions. Up to 140 °C, the DSC curves of nov_water and res_nov are virtually indistinguishable, which proves that the initial stage of cross-linking proceeds by the same, water-enabled mechanism in both systems. The res_nov mixture exhibits an additional exothermic effect at a higher temperature (∼180–210 °C), which can be attributed to curing via hydroxymethylene groups. This confirms that, while HMTA-mediated cross-linking is initiated earlier in the presence of water, resole-specific condensation reactions proceed independently at higher temperatures.

In the phoh_hmta system, sealing the crucible suppresses phenol evaporation and reveals an exothermic reaction beginning at 146 °C, with the exotherm maximum at 162 °C. This demonstrates that phenol is, in fact, capable of reacting with HTMA when volatilization is prevented. Due to the high concentration of HMTA, multiple intermediates were also formed during the first reaction. Importantly, however, the curing temperature remains significantly higher than that in nov_water or res_nov, proving that phenol alone is not responsible for the strong temperature shift observed in resole-containing systems.

All transitions observed during high-pressure DSC measurements are listed in Table 3.

**Table 3:** List of All Transitions Observed in High-Pressure DSC Measurements

sample	thermally induced process	onset [°C]	peak [°C]
res_nov steel	cross-linking with HMTA	118	140 exo
	curing with hydroxymethylene		∼180 exo
	decomposition of intermediates		∼210 exo
nov_water steel	cross-linking with HMTA	117	140 exo
	water evaporation (probably unsealed crucible)		194 endo
	decomposition of intermediates		223 exo
nov steel	cross-linking with HMTA	137	149 exo
	decomposition of intermediates (slightly pronounced)		∼226 exo
	glass transition		∼70
nov Al	cross-linking with HMTA	143	151 exo
	melting of phenol		43 endo
	unassigned		97–107 mixed
phoh_hmta steel	curing (polymerization) with HMTA	146	162 exo
	decomposition of intermediates		216 exo

Together, these results provide direct experimental evidence that water is the key factor responsible for lowering the curing temperature of phenolic systems cross-linked with HMTA. The presence of water, either added explicitly or generated during resole curing, initiates HMTA hydrolysis, producing reactive intermediates that trigger cross-linking at significantly lower temperatures.

## 4 Discussion

Curing of novolac resins with hexamethylenetetramine is known to initiate rapidly above 150 °C, with a well-defined exothermic peak and the evolution of ammonia as a characteristic byproduct. In contrast, resole resins cure over a broad temperature range, driven by hydroxymethylene condensation reactions accompanied by water formation and volatilization of low-molecular components. When these two resin types are combined, their curing behavior does not represent a simple superposition of two independent processes.

The present results demonstrate that simultaneous curing of novolac and resole in the presence of HMTA leads to a pronounced shift of the cross-linking reaction toward lower temperatures. In the mixture, the onset of ammonia evolution occurs at approximately 130 °C, compared to ∼157 °C for novolac alone, and the main exothermic peak is shifted by about 10 °C. At the same time, the emission profile of ammonia is altered: the early stage release is suppressed, whereas the secondary emission at elevated temperatures becomes more pronounced. This behavior indicates that the reaction pathway is modified and favors the formation of semistable nitrogen-containing intermediates.

Two classes of factors may account for this shift: (i) the presence of low-molecular-weight phenolic products acting as plasticizers and (ii) chemical interactions introduced by hydroxymethylene groups and water. The model systems decisively differentiate between these possibilities. Phenol mixed with HMTA shows no significant curing under open conditions and exhibits massive volatilization. Only when evaporation is suppressed in high-pressure crucibles does phenol participate in HMTA-mediated reactions, and even then, the curing temperature remains comparable to that of novolac. This excludes phenol as the primary cause of the observed temperature shift.

In contrast, both resole–HMTA and salicyl alcohol–HMTA systems exhibit early ammonia evolution and exothermic curing in the same temperature range as for the resole–novolac mixture. These systems share a common feature—the presence of hydroxymethylene groups, which generate water during condensation reactions. Although no direct reaction pathway between hydroxymethylene groups and HMTA has been reported, HMTA is known to undergo hydrolysis in the presence of water, producing formaldehyde and aminomethyl species capable of rapidly reacting with phenolic rings.

High-pressure DSC provides direct evidence of this mechanism. The addition of only 5 wt% of water to novolac shifts the onset and peak temperature of curing to the same values observed for the resole–novolac mixture. Moreover, even the small amount of moisture inherently present in commercial novolac measurably affects the curing temperature when retained in a sealed crucible. These results demonstrate that water alone is sufficient to initiate the low-temperature curing pathway.

In the resole–novolac system, water is both initially present and continuously generated during hydroxymethylene-driven curing. As a consequence, HMTA is hydrolyzed already at relatively low temperatures, producing reactive intermediates that initiate cross-linking earlier than in neat novolac. At higher temperatures, resole-specific condensation reactions proceed in parallel, resulting in a complex, multistage curing process. The enhanced secondary ammonia emission observed for mixtures reflects the accumulation and subsequent decomposition of nitrogen-containing intermediates formed under these conditions.

The curing of hybrid phenolic systems is therefore governed by a coupled mechanism: HMTA-mediated cross-linking is accelerated by water, while hydroxymethylene condensation remains active at elevated temperatures. The two pathways interact through shared reactants and intermediates, making the process highly sensitive to the moisture content, resin composition, and processing conditions.

A key outcome of this study is the identification of water as the primary mediator of these interactions. While novolac cured with HMTA alone follows the well-established cross-linking pathway described in the literature, the presence of resole introduces both physically bound water and water generated in situ during curing via hydroxymethylene groups. This additional water enables early hydrolysis of HMTA, generating reactive formaldehyde and aminomethyl intermediates that participate in cross-linking reactions at lower temperatures than in water-deficient systems. Such a mechanism, previously proposed in literature,^22,42^ is here supported experimentally by the observed shifts in curing behavior.

The results obtained for the reference and model systems provide strong evidence against a direct chemical reaction between HMTA and hydroxyl groups as the dominant mechanism responsible for the temperature shift. Phenol–HMTA mixtures, despite containing phenolic hydroxyl groups, do not undergo appreciable cross-linking under open-crucible conditions, indicating that phenol itself does not activate HMTA effectively. In contrast, both resole–HMTA and salicyl alcohol–HMTA systems exhibit early ammonia evolution and curing-related thermal effects, which point to an indirect role of hydroxymethylene chemistry. This role is best rationalized by the production and retention of water rather than by direct covalent interactions between HMTA and hydroxymethylene moieties. The curing behavior of resole–novolac mixtures further supports this interpretation. Although their overall thermal profiles resemble those of novolac, the presence of resole shifts the onset of HMTA-related reactions and modifies the balance between HMTA consumption and volatilization. As a consequence, a larger fraction of nitrogen-containing intermediates appears to survive the primary curing stage and decomposes at higher temperatures, leading to secondary ammonia evolution. This behavior contrasts with novolac cured alone, where HMTA consumption and network formation are more tightly confined to a narrower temperature range. The effect of water on curing time for novolac resins was previously studied by Tonogai et al., with the use of solvent extraction method.^43^

The influence of minor pH modifications on the curing process is secondary to the effect of water but provides additional insight into the complexity of HMTA activation. The observed differences between acid- and base-modified systems suggest that multiple initiation pathways, such as hydrogen bonding between HMTA and phenolic hydroxyl groups and HMTA hydrolysis, may operate concurrently. Small changes in pH can shift their relative contributions without fundamentally changing the dominant curing mechanism. This explains why pH additives modify the kinetics but do not eliminate the temperature shift induced by the presence of a resole.

High-pressure DSC experiments provide decisive confirmation of the water-mediated mechanism. When evaporation is suppressed, and water is retained within the system, novolac exhibits curing behavior essentially identical with that of resole–novolac mixtures. This demonstrates that water alone is sufficient to reproduce the temperature shift, independent of other structural features of the resole. In open systems, however, only water that is physically bound or generated during hydroxymethylene-driven reactions remains available long enough to influence HMTA chemistry, explaining why the effect is particularly pronounced in resole-containing formulations.

From a practical perspective, these findings highlight the critical role of water in the curing of phenolic resins with HMTA. Water originating from resin composition, fillers, environmental humidity, or curing reactions can significantly alter the curing kinetics and reaction pathways. In mixed resole–novolac systems, complete elimination of water is neither realistic nor desirable, and its presence must therefore be treated as an intrinsic parameter of the curing process. This has important implications for the design, optimization, and reproducibility of industrial formulations, particularly in abrasive and friction materials, where such resin systems are commonly employed.

## 5 Conclusions

This study demonstrates that simultaneous curing of novolac and resole resins in the presence of hexamethylenetetramine fundamentally alters the cross-linking behavior of phenolic systems. Compared to the curing of individual resins, the hybrid system exhibits:

- a significant shift of the curing reaction toward lower temperatures,
- an earlier onset of ammonia evolution,
- a modified emission profile indicating the formation of thermally labile nitrogen-containing intermediates.

Model compound experiments show that this effect cannot be attributed to the presence of low-molecular phenols alone. Instead, hydroxymethylene-containing structures characteristic of resoles play a decisive role.

High-pressure DSC measurements provide direct proof that water is the key factor responsible for the temperature shift; i.e., addition of only 5 wt% of water to novolac reproduces the curing behavior of the resole–novolac mixture, and even trace moisture present in commercial novolac affects curing when retained in the reaction system.

These findings indicate that water initiates an alternative curing pathway by promoting hydrolysis of HMTA, generating reactive formaldehyde and aminomethyl intermediates that trigger cross-linking at lower temperatures. In hybrid systems, water is continuously produced during resole curing, making this mechanism unavoidable.

From a technological perspective, the results highlight that moisture content, originating from raw materials, processing environment, or in situ reactions, has a profound impact on the kinetics and mechanism of phenolic resin curing. This must be considered in the design of resin formulations, processing windows, and thermal schedules for phenolic-based composites, particularly in applications such as abrasive and friction materials, where simultaneous curing of resole and novolac is standard practice.

## Supplementary Material



## Notes

DSC curves obtained using NETZSCH DSC 214 Polyma, not analyzed in this study, including neat novolac, novolac with 14% HMTA, neat HMTA, and resole (PDF)

## Abbreviations

HMTA: hexamethylenetetramine (common curing agent for novolac resins)
DSC: differential scanning calorimetry
FTIR: Fourier transform infrared spectroscopy
PF: phenol–formaldehyde­(resin)
NMR: nuclear magnetic resonance
STA: simultaneous thermal analysis
TG: thermogravimetric
DTG: Derivative Thermogravimetric
