# Supplementary material for: Interaction of Hexamethylenetetramine with Phenol–Formaldehyde Resin during Simultaneous Curing of Novolac and Resole
Source: ACS Omega. 2026 May 1;11(18):26206–19. doi: 10.1021/acsomega.5c09518 (PMC13177034; doi:10.1021/acsomega.5c09518)
Supplement: Supplementary file 1 [file ao5c09518_si_001.pdf]

Interaction of hexamethylenetetramine with phenol-formaldehyde resin during simultaneous curing of novolac and resole

Bartłomiej Milewski [1,2], Robert Antosz[2], Marcin Skowronek[3] and Jadwiga Laska [1]**

AGH University of Krakow, Faculty of Materials Science and Ceramics, Al. Mickiewicza 30, 30-059 Kraków, Poland;

B.M. milewski@agh.edu.pl, J.L. jlaska@agh.edu.pl

TECHNIFLEX Sp. z o.o., Company Branch in Mszana Dolna, ul. Spadochroniarzy 8, 34-730 Mszana Dolna, Poland; robert.antosz@techniflex.pl

Lerg S.A., Pustków – Osiedle 59D, 39-206 Pustków 3, Poland; marcin.skowronek@lerg.pl

* Correspondence: B.M. milewski@agh.edu.pl, J.L. jlaska@agh.edu.pl

Supporting information – additional DSC curves obtained using NETZSCH DSC 214 Polyma at 10K/min heating rate:

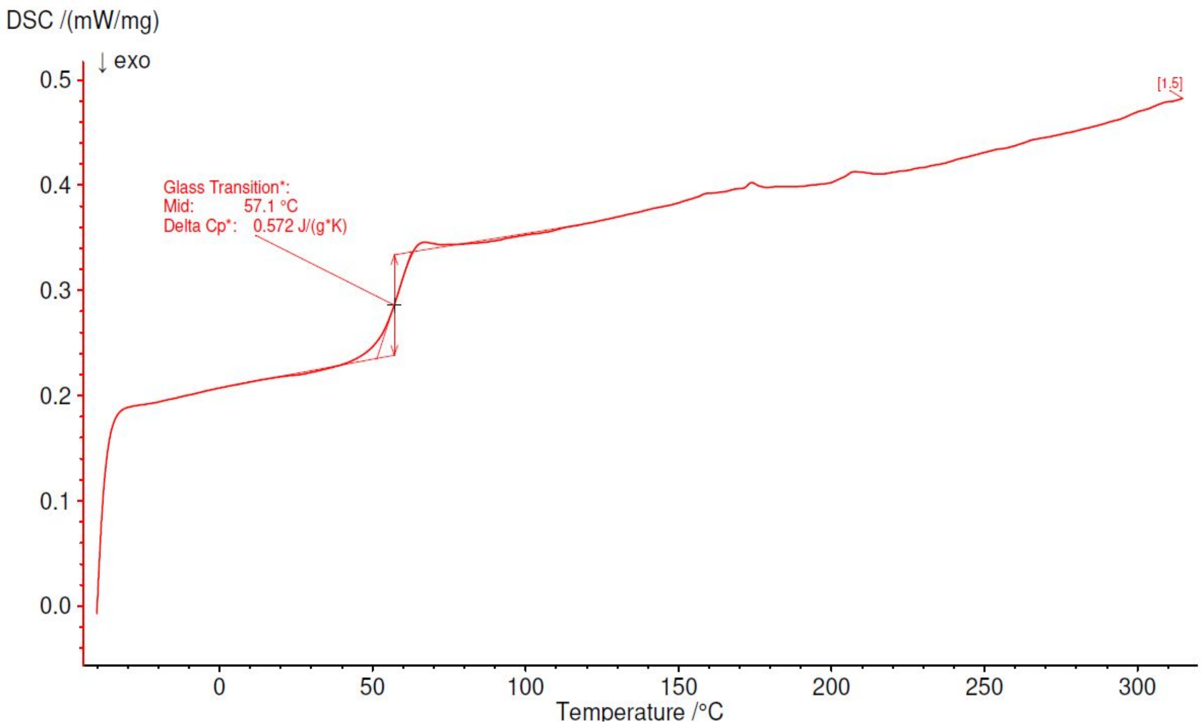

Figure S1- DSC curve for neat novolac (without curing agent) in a closed pierced aluminum crucible. Only glass transition in present.

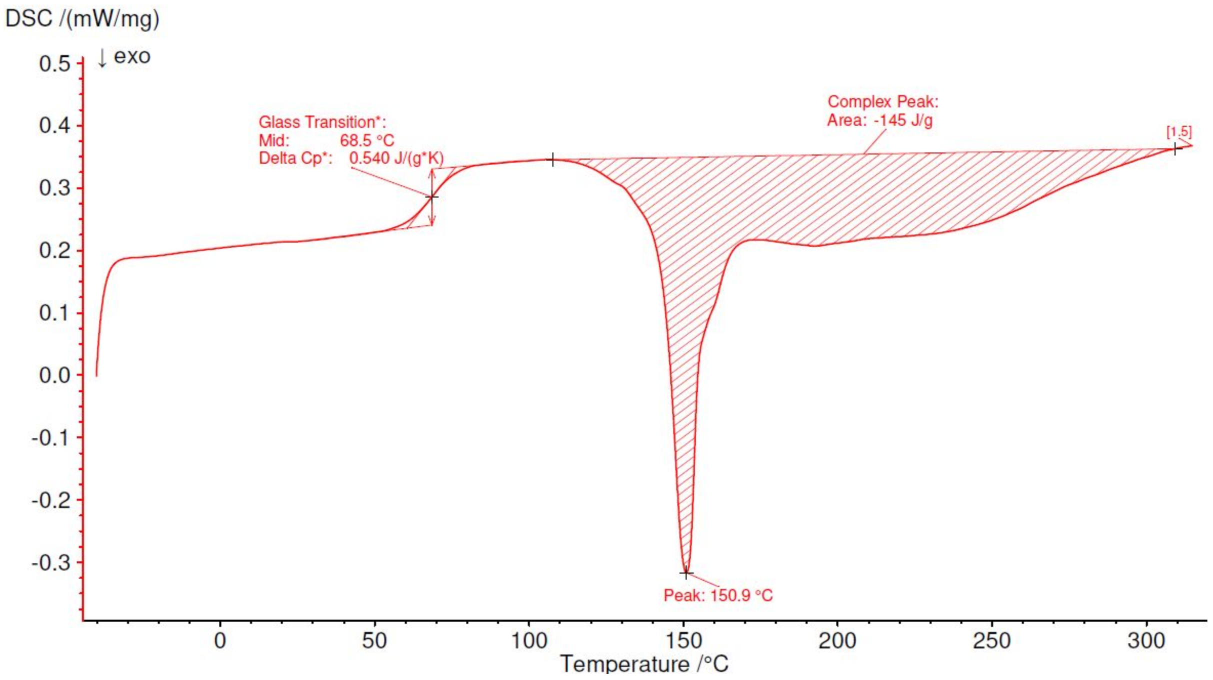

Figure S2 - DSC curve for sample nov (novolac containing 14% HMTA) in a closed pierced aluminum crucible. Both glass transition and exothermic curing peak are present.

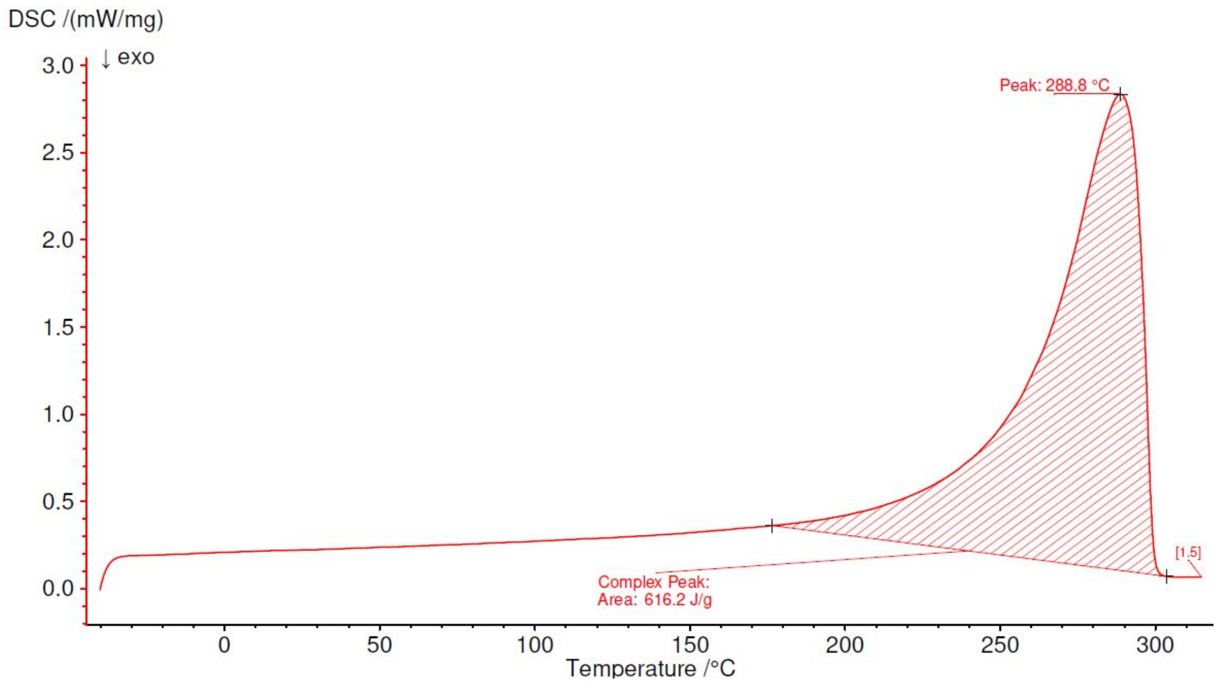

Figure S3 - DSC curve for HMTA in a closed pierced aluminum crucible. No transitions other than sublimation are present.

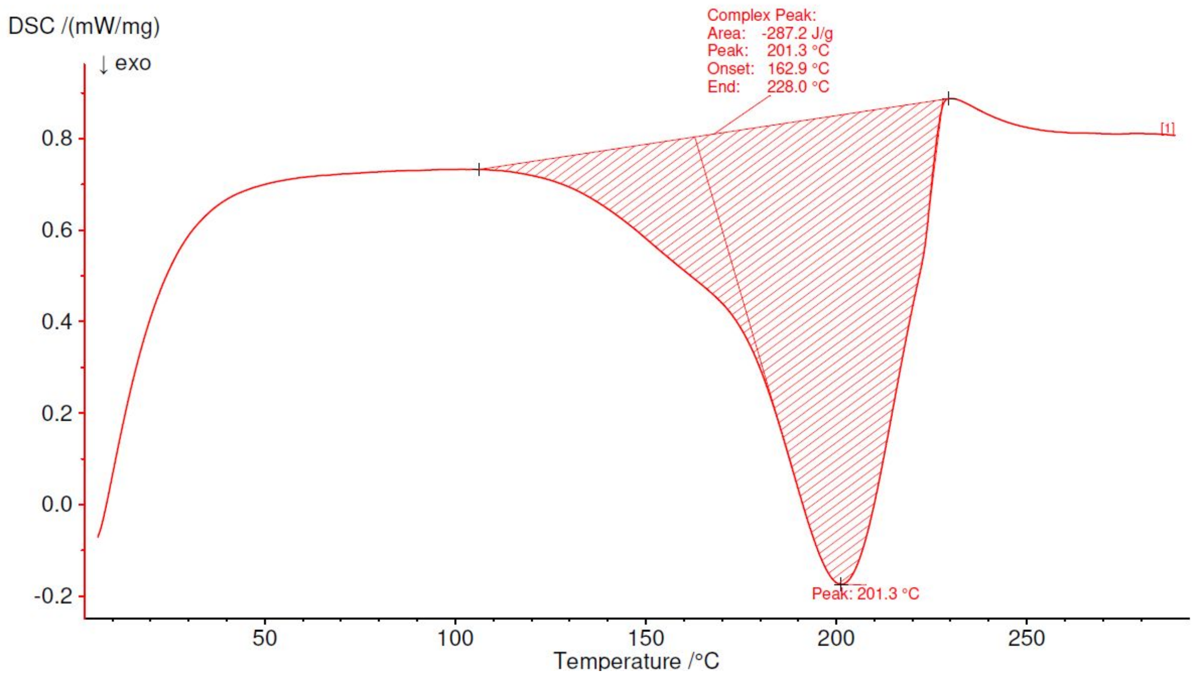

Figure S4 - DSC curve for sample res (resole). High-pressure steel crucible was used. Only exothermic peak characteristic for curing is present.
